# Characteristics of serum Metrnl levels in patients with neuromyelitis optica spectrum disorder and their associations with disease activity and inflammatory biomarkers

**DOI:** 10.3389/fimmu.2026.1957990

**Published:** 2026-10-13

**Authors:** Li-Min Li, Yingzhe Shao, Hao Cai, Li Yang

**Affiliations:** 1 Department of Neurology, Tianjin Neurological Institute, Tianjin Medical University General Hospital, Tianjin, China; 2 Department of Neurology, Tianjin Huanhu Hospital, Tianjin, China

**Keywords:** biomarker, disease activity, inflammatory cytokines, Metrnl, neuromyelitis optica spectrum disorder

## Abstract

**Background:**

Neuromyelitis optica spectrum disorder (NMOSD) is a severe autoimmune inflammatory disease for which reliable biomarkers of disease activity are lacking. Meteorin-like protein (Metrnl) has not been investigated in NMOSD; this study assessed its clinical significance in NMOSD patients.

**Methods:**

Serum Metrnl, interleukin (IL)-6, and IL-17A levels were quantified by enzyme-linked immunosorbent assay in 50 patients with NMOSD and 30 age- and sex-matched healthy controls (HCs) and correlated with clinical and laboratory data. Metrnl levels were also remeasured in 30 patients before and after high-dose methylprednisolone therapy, together with derived inflammatory indices. Benjamini–Hochberg false-discovery-rate (FDR) correction was applied within prespecified test families, with a sensitivity power analysis. Metrnl alone and a combined multimarker logistic regression model were evaluated by receiver operating characteristic (ROC) analysis, DeLong tests, bootstrap validation, calibration, and decision curve analysis.

**Results:**

Serum Metrnl levels were higher in NMOSD patients than in HCs and were greater in those with mild disability than in those with moderate-to-severe disability (both *p* < 0.001); the increase in patients with gadolinium-enhancing lesions was nominal (*p* = 0.034, *q* = 0.068). After FDR correction, serum Metrnl levels correlated negatively with IL-6, IL-17A, neutrophil-to-lymphocyte ratio (NLR), systemic immune-inflammation index (SII), and Expanded Disability Status Scale (EDSS) score (all *q* < 0.05), but not with high-sensitivity C-reactive protein (hs-CRP). Metrnl alone distinguished NMOSD patients from HCs (area under the curve [AUC] 0.752), whereas a combined model of Metrnl, IL-6, IL-17A, and hs-CRP performed markedly better (AUC 0.933; DeLong *p* < 0.001), increasing specificity from 53.3% to 80.0% at 100% sensitivity. After methylprednisolone therapy, Metrnl, IL-6, and IL-17A levels decreased (*p* < 0.001) along with clinical improvement, whereas NLR, platelet-to-lymphocyte ratio, and SII remained unchanged; the decrease in Metrnl levels (median −25.5%) was the largest and was uncorrelated with changes in other biomarkers.

**Conclusions:**

Serum Metrnl levels were elevated in patients with NMOSD, inversely related to disease severity and inflammatory biomarkers, and decreased after immunotherapy, supporting it as a dynamic biomarker of disease activity and a component of a multimarker diagnostic panel. The cross-sectional design precludes causal inference, and exploratory subgroup findings require external validation.

## 1 Introduction

Neuromyelitis optica spectrum disorder (NMOSD) is a severe autoimmune-mediated inflammatory disorder of the central nervous system (CNS), which predominantly affects the optic nerves and spinal cord (1). The precise underlying pathogenic mechanisms remain incompletely understood. Current evidence indicates that NMOSD is primarily driven by pathogenic autoantibodies against aquaporin-4 (AQP4)—a water channel protein densely expressed on astrocytic endfeet—together with AQP4-specific T helper 17 (Th17) cells (2, 3). Disease initiation occurs in peripheral immune compartments (including lymph nodes and bone marrow), where autoreactive lymphocytes undergo activation and differentiation. These primed cells subsequently breach the blood–brain barrier (BBB) and infiltrate the CNS parenchyma, thus amplifying neuroinflammation through proinflammatory cytokine networks, complement activation, and the recruitment of monocytes and neutrophils (4–6). Histopathologically, NMOSD reflects the coordinated action of multiple immune effector pathways, culminating in primary astrocyte injury, secondary demyelination, oligodendrocyte apoptosis, and axonal damage (7–9). Although the full pathogenic cascade remains incompletely delineated, dysregulated neuroinflammatory responses constitute the core pathological driver of disease progression. Accumulating evidence underscores the pivotal role of cytokine-mediated immune dysregulation in NMOSD pathogenesis, thereby reinforcing the potential utility of cytokines as both prognostic biomarkers and therapeutically actionable targets (10–12). Nevertheless, early intervention in NMOSD remains clinically challenging owing to diagnostic delays and phenotypic heterogeneity; thus, the timely initiation of high-efficacy immunosuppressive therapy is essential to mitigate acute neuroinflammation and prevent irreversible neurological sequelae. Consequently, the discovery and validation of novel molecular biomarkers and mechanistically informed therapeutic targets are imperative for improving long-term neurological outcomes in patients with NMOSD.

Meteorin-like protein (Metrnl), which is also known as Meteorin-β, Subfatin, interleukin (IL)-41, and Cometin, is a recently identified multifunctional cytokine involved in the regulation of diverse physiological and pathological processes, including inflammatory and immune responses, energy metabolism, and tissue homeostasis (13). In both humans and mice, Metrnl is preferentially expressed in skeletal muscle, barrier tissues (e.g., skin epidermis and intestinal mucosa), and M2-polarized macrophages (14). Subsequent studies have demonstrated that Metrnl is secreted by multiple innate immune cell types, including distinct macrophage subsets, dendritic cells, and select granulocyte populations; however, it is undetectable in lymphoid lineages, specifically CD4^+^ and CD8^+^ T cells, B cells, natural killer cells, and natural killer T cells (15). Metrnl-knockout mice exhibit impaired B-cell development and reduced serum IgG levels, indicating that Metrnl plays a role in maintaining humoral immune homeostasis (16). Clinically, Metrnl expression is elevated in the synovial tissue and peripheral blood of patients with rheumatoid arthritis (RA) and correlates positively with disease activity (17); similarly, serum Metrnl levels are significantly increased in patients with myasthenia gravis (MG) and are associated with clinical severity (18). *In vitro* and *in vivo* studies have further demonstrated that Metrnl expression in bone marrow–derived cells is inducible by proinflammatory stimuli and that Metrnl itself can modulate the production of key immunoregulatory cytokines implicated in NMOSD pathogenesis (particularly IL-6 and IL-17A) (19, 20). Nevertheless, the functional role and clinical relevance of Metrnl in NMOSD remain incompletely characterized.

In this study, serum Metrnl levels were quantified in patients with NMOSD, and their associations with clinical phenotypes and other inflammatory biomarkers were assessed to explore the potential pathophysiological relevance of Metrnl in NMOSD.

## 2 Materials and methods

### 2.1 Recruitment of patients and controls

A total of 50 patients with NMOSD experiencing an acute attack were consecutively enrolled from the Department of Neurology, Tianjin Medical University General Hospital, between January 2023 and February 2026. Thirty healthy controls (HCs), matched to the NMOSD group for age and sex, were recruited concurrently. All patients met the 2015 International Consensus Diagnostic Criteria for NMOSD (21). The following exclusion criteria were applied: 1) active infection within 3 months prior to enrollment; 2) coexisting autoimmune disorders or malignancies; 3) pre-existing hyperlipidemia, diabetes mellitus, cerebrovascular disease, coronary heart disease, or cognitive impairment; and 4) use of systemic corticosteroid or immunosuppressant therapy (including azathioprine, cyclophosphamide, or mycophenolate mofetil) within 4 weeks before enrollment, or B-cell–depleting therapy within 6 months before enrollment. Baseline blood samples were collected on the day of admission, before the first dose of high-dose intravenous methylprednisolone, and all patients subsequently received the same acute treatment, namely high-dose intravenous methylprednisolone pulse therapy. This study was conducted in strict accordance with the ethical principles of the Declaration of Helsinki and was approved by the Medical Ethics Committee of Tianjin Medical University General Hospital (Approval No. IRB2022-KY-264). Written informed consent was obtained from all participants before enrollment.

### 2.2 Clinical and laboratory assessments

Systematic demographic and clinical data were collected from all participants. Height and body weight were measured in light clothing without shoes, and body mass index (BMI) was calculated as weight in kilograms divided by the square of height in meters. Laboratory assessments involved measurements of complete blood count parameters, including white blood cell (WBC), neutrophil, lymphocyte, monocyte, and platelet counts, as well as high-sensitivity C-reactive protein (hs-CRP), blood glucose, triglyceride (TG), total cholesterol (TC), low-density lipoprotein cholesterol (LDL-C), and high-density lipoprotein cholesterol (HDL-C) levels. Derived inflammatory indices were calculated as follows: neutrophil-to-lymphocyte ratio (NLR), platelet-to-lymphocyte ratio (PLR), and monocyte-to-lymphocyte ratio (MLR) were defined as the respective absolute counts divided by the absolute lymphocyte count; WBC-to-HDL-C ratio (WHR), neutrophil-to-HDL-C ratio (NHR), and monocyte-to-HDL-C ratio (MHR) were computed by dividing the corresponding cellular counts (WBCs, neutrophils, or monocytes) by the serum HDL-C level; systemic immune-inflammation index (SII) was calculated as the product of platelet and neutrophil counts divided by the lymphocyte count; and systemic inflammation response index (SIRI) was calculated as the product of neutrophil and monocyte counts divided by the lymphocyte count. Neurological disability was independently evaluated by two neurologists using the Expanded Disability Status Scale (EDSS), and the CNS lesion burden was evaluated via magnetic resonance imaging (MRI). Patients were stratified into two groups based on disability severity: the mild disability (EDSS score < 4) and moderate/severe disability (EDSS score ≥ 4) groups (22). Additionally, patients with NMOSD were categorized according to age at disease onset into early-onset NMOSD (EO-NMOSD; onset age < 50 years) and late-onset NMOSD (LO-NMOSD; onset age ≥ 50 years) groups (23). An EDSS score of 4.0 was adopted as the cutoff for two reasons. First, it is the point at which the scale changes construct: steps below 4.0 are defined by the functional-system scores of patients who remain fully ambulatory, whereas a score of 4.0 denotes relatively severe disability that begins to limit full daily activity in a patient who remains self-sufficient, is up and about for approximately 12 hours per day, and can walk approximately 500 m without aid or rest. Second, an EDSS score of 4.0 is an established disability milestone in NMOSD, having been used both as an endpoint in natural-history analyses of disability accumulation (23) and as the threshold separating mild from moderate-to-severe disability in previous biomarker studies of this disease (22); adopting the same threshold ensures that the present results are directly comparable to those in the literature.

### 2.3 Serum Metrnl, inflammatory cytokines, and AQP4-IgG detection

Venous blood samples were drawn from all participants in the early morning following an overnight fast of at least 8 hours. In a subset of 30 patients with NMOSD, paired serum samples were obtained before and after high-dose intravenous methylprednisolone therapy. Serum was isolated via centrifugation at 1000 × g for 10 min at 4 °C, immediately aliquoted into sterile polypropylene tubes, and stored at −80 °C until analysis. Serum levels of Metrnl, IL-6, and IL-17A were quantified using commercially available, analytically validated ELISA kits (Wuhan Fine Biotech Co., Ltd.), strictly according to the manufacturer’s instructions. All the assays were performed in duplicate under double-blind conditions. Serum AQP4-IgG status in patients with NMOSD was determined by using a live cell–based assay.

### 2.4 Statistical analysis

Statistical analyses were conducted using IBM SPSS Statistics version 26.0, GraphPad Prism version 8.0, and R version 4.4.1. The Shapiro–Wilk test was used to evaluate the normality of continuous variables. Categorical variables were summarized as frequencies and percentages and compared using the chi-square test or Fisher’s exact test, as appropriate. Continuous variables were presented as the mean ± standard deviation (SD) for normally distributed data and were analyzed by Student’s *t*-test; for nonnormally distributed data, medians with interquartile ranges (IQRs) were reported, and group comparisons were performed using the Mann–Whitney U test. Paired pre- and post-treatment measurements were compared using the paired *t*-test or the Wilcoxon signed-rank test according to the distribution of the within-patient differences, and standardized effect sizes (Cohen’s *d* for independent samples, *d*^z^ for paired samples) were reported. Treatment-associated changes were additionally expressed as the median percentage change from baseline, and the relationships between the changes in serum Metrnl levels and the concurrent changes in IL-6, IL-17A, NLR, PLR, and SII were assessed by Spearman’s rank correlation coefficient of the paired differences. Multivariable logistic regression was used to identify factors that were independently associated with NMOSD, and odds ratios (ORs) with 95% confidence intervals (CIs) were calculated per 1-SD increment for continuous variables. To limit overfitting, no more than four predictors were retained. The predicted probability derived from the final model was used as the combined diagnostic index. Receiver operating characteristic (ROC) curve analysis was performed to assess the diagnostic performance of serum Metrnl in differentiating patients with NMOSD from HCs. Differences between the AUCs were assessed by the DeLong test. The incremental value of Metrnl over the remaining biomarkers was further evaluated by the likelihood-ratio test, the integrated discrimination improvement (IDI), and the continuous net reclassification improvement (NRI), with 95% CIs derived from 2000 bootstrap resamples. The combined model was internally validated by bootstrapping (1000 resamples) to obtain an optimism-corrected AUC and by repeated fivefold cross-validation (200 repetitions). Calibration was assessed by the Hosmer–Lemeshow goodness-of-fit test, the Brier score and a bootstrap bias-corrected calibration curve, and clinical utility was examined by decision curve analysis. Logistic regression modeling, model validation, decision curve analysis and nomogram construction were performed in R. Correlations between serum Metrnl levels and clinical or laboratory parameters were evaluated using Spearman’s rank correlation coefficient for nonnormally distributed variables and Pearson’s correlation coefficient for normally distributed variables. Because multiple clinical and laboratory parameters were examined, all *p* values were additionally corrected for multiplicity using the Benjamini–Hochberg false-discovery-rate (FDR) procedure (24) within four prespecified families of tests: (i) the 25 NMOSD-versus-HC comparisons summarized in **Table 1**, (ii) the four comparisons of serum Metrnl across clinical subgroups of NMOSD, (iii) the 15 correlations between serum Metrnl and clinical or laboratory parameters, and (iv) the seven paired pre- versus post-treatment comparisons. Both unadjusted *p* values and FDR-adjusted *q* values were reported, and findings were described as significant only when *q* < 0.05. No *a priori* sample-size calculation was performed because the cohort comprised all consecutively eligible patients who were recruited during the study period; a sensitivity power analysis was therefore conducted to define the effect sizes detectable with 80% power at a two-sided α of 0.05, and FDR correction and power analyses were carried out in R. To determine whether BMI confounded the relationship between serum Metrnl and disability, a multivariable linear regression model was fitted with serum Metrnl as the dependent variable and EDSS score, BMI, age, and sex as independent variables, and partial correlation coefficients between serum Metrnl and EDSS score were calculated before and after adjustment for BMI. A two-tailed *p* value < 0.05 was considered statistically significant.

**Table 1:** Demographic and clinical characteristics of the study participants.

Variables	NMOSD (n = 50)	HC (n = 30)	*p*	*q*
Sex, F/M	42/8	27/3	0.451	0.773
Age, y	52.68 ± 15.32	53.57 ± 15.37	0.803	0.958
BMI (kg/m²)	23.13 ± 3.04	23.48 ± 2.72	0.609	0.902
Disease duration, median (IQR), months	11.5 (6.0–21.0)	–	–	–
EDSS scores	4.35 ± 2.02	–	–	–
Number of relapses, median (IQR)	1 (1–2)	–	–	–
Annualized relapse rate, median (IQR)	1.37 (0.82–2.40)	–	–	–
Type of attack, n (%)	50 (100%)			–
Optic neuritis	16 (32%)	–	–	–
Myelitis	39 (78%)	–	–	–
Area postrema syndrome	3 (6%)	–	–	–
Other types	6 (12%)	–	–	–
Laboratory test results				–
TG (mmol/L)	1.11 ± 0.33	1.22 ± 0.35	0.186	0.343
TC (mmol/L)	4.15 ± 0.83	4.24 ± 0.74	0.613	0.920
LDL-C (mmol/L)	2.53 ± 0.58	2.30 ± 0.63	0.105	0.210
HDL-C (mmol/L)	1.17 ± 0.28	1.17 ± 0.33	0.963	0.963
FBG (mmol/L)	5.14 ± 0.62	5.24 ± 0.69	0.503	0.805
WBC, median (IQR), (10^9^/L)	6.68 (5.68–8.93)	6.64 (5.47–8.57)	0.735	0.958
NEU, median (IQR), (10^9^/L)	5.37 (3.33–7.07)	3.83 (3.13–5.03)	0.020	0.059
LYM, median (IQR), (10^9^/L)	1.63 (1.36–2.25)	1.66 (1.34–2.15)	0.921	0.961
MON, median (IQR), (10^9^/L)	0.44 (0.33–0.61)	0.44 (0.35–0.65)	0.878	0.958
PLT, median (IQR), (10^9^/L)	280.00 (238.50–330.50)	232.00 (160.00–294.25)	**0.002**	**0.008**
NLR, median (IQR)	3.05 (1.71–4.64)	2.06 (1.50–2.93)	0.022	0.059
MLR, median (IQR)	0.25 (0.20–0.40)	0.25 (0.20–0.35)	0.854	0.958
PLR, median (IQR)	172.27 (121.51–210.09)	131.07 (96.15–162.91)	**0.009**	**0.031**
SII, median (IQR)	792.75 (591.87–1355.76)	487.11 (346.39–674.49)	**<0.001**	**0.002**
SIRI, median (IQR)	1.48 (0.82–2.80)	0.99 (0.69–1.63)	0.097	0.210
WHR, median (IQR)	6.03 (4.71–8.21)	5.73 (4.97–8.04)	0.766	0.958
NHR, median (IQR)	4.48 (3.21–6.25)	3.38 (2.59–4.95)	0.074	0.178
MHR, median (IQR)	0.42 (0.27–0.55)	0.44 (0.24–0.61)	0.874	0.958
hs-CRP (mg/L)	2.39 (1.34–4.78)	1.23 (0.83–2.43)	**<0.001**	**0.002**

NMOSD, neuromyelitis optica spectrum disorder; HC, healthy control; EDSS, Expanded Disability Status Scale; n, number; y, year; F, female; M, male; IQR, interquartile range; TG triglyceride; TC total cholesterol; LDL-C, low-density lipoprotein cholesterol; HDL-C, high-density lipoprotein cholesterol; BMI, body mass index; FBG, fasting blood glucose; WBC, white blood cell; NEU, neutrophil; LYM, lymphocyte; MON, monocyte; PLT, platelet; NLR, neutrophil-to-lymphocyte ratio; PLR, platelet-to-lymphocyte ratio; MLR, monocyte-to-lymphocyte ratio; WHR, WBC-to-HDL-C ratio; NHR, neutrophil-to-HDL-C ratio; MHR, monocyte-to-HDL-C ratio; SII, systemic immune-inflammation index; SIRI, systemic inflammation response index; hs-CRP, high-sensitivity C-reactive protein. *q*, Benjamini–Hochberg false-discovery-rate–adjusted *p* values are shown for the 25 NMOSD-versus-HC comparisons in this family (22 variables, along with Metrnl, IL-6, and IL-17A); bold denotes *q* < 0.05.

## 3 Results

### 3.1 Demographic and clinical characteristics of all participants

The baseline demographic and clinical characteristics of patients with NMOSD and HCs are summarized in **Table 1**. A total of 50 patients with NMOSD and 30 HCs were enrolled. All the patients with NMOSD were seropositive for AQP4-IgG. No significant differences in age (*p* = 0.803) or sex (*p* = 0.451) were observed between the two groups. BMI was also comparable between patients with NMOSD (23.13 ± 3.04 kg/m^2^) and HCs (23.48 ± 2.72 kg/m^2^; *p* = 0.609, *q* = 0.902; **Table 1**). Among the NMOSD cohort, 19 patients were classified as EO-NMOSD, whereas the remaining 31 patients were classified as LO-NMOSD. Additionally, 27 patients presented with mild disability (EDSS score < 4), whereas 23 patients exhibited moderate-to-severe disability (EDSS score ≥ 4). The median disease duration at enrollment was 11.5 months (IQR: 6.0–21.0; range: 2–48 months), the median number of attacks was 1 (IQR: 1–2), and the median annualized relapse rate was 1.37 (IQR: 0.82–2.40).

### 3.2 Serum levels of Metrnl and other inflammatory biomarkers in NMOSD

Serum Metrnl levels were significantly greater in patients with NMOSD (1298.11 ± 483.87 pg/mL) than in HCs (832.89 ± 470.82 pg/mL; *p* < 0.001, *q* < 0.001; **Figure 1A**). Sex had no significant effect on Metrnl expression (*p* = 0.331, *q* = 0.441; **Figure 1B**). Patients with MRI-enhancing lesions on brain or spinal cord imaging had higher serum Metrnl levels (1410.76 ± 522.96 pg/mL) than those without such lesions (1114.30 ± 352.17 pg/mL; Cohen’s *d* = 0.64; **Figure 1C**); this difference was nominally significant (*p* = 0.034) but did not survive FDR correction (*q* = 0.068) and is therefore reported as exploratory. Consistent with these findings, the sensitivity power analysis indicated that the available subgroup sizes (n = 31 versus n = 19) afforded only 57% power to detect an effect of the observed magnitude, whereas the primary NMOSD-versus-HC comparison (n = 50 versus n = 30) had ≥ 98% power (minimum detectable *d* = 0.66). Notably, patients with mild disability (EDSS score < 4) had significantly higher serum Metrnl levels (1506.31 ± 414.44 pg/mL) than those with moderate-to-severe disability (EDSS score ≥ 4) (1053.70 ± 450.37 pg/mL; *p* < 0.001, *q* = 0.002, Cohen’s *d* = 1.05; **Figure 1D**). Similarly, no significant difference was observed between patients with EO-NMOSD (1357.99 ± 586.67 pg/mL) and those with LO-NMOSD (1261.40 ± 415.01 pg/mL; *p* = 0.535, *q* = 0.535; **Figure 1E**).

**Figure 1:**
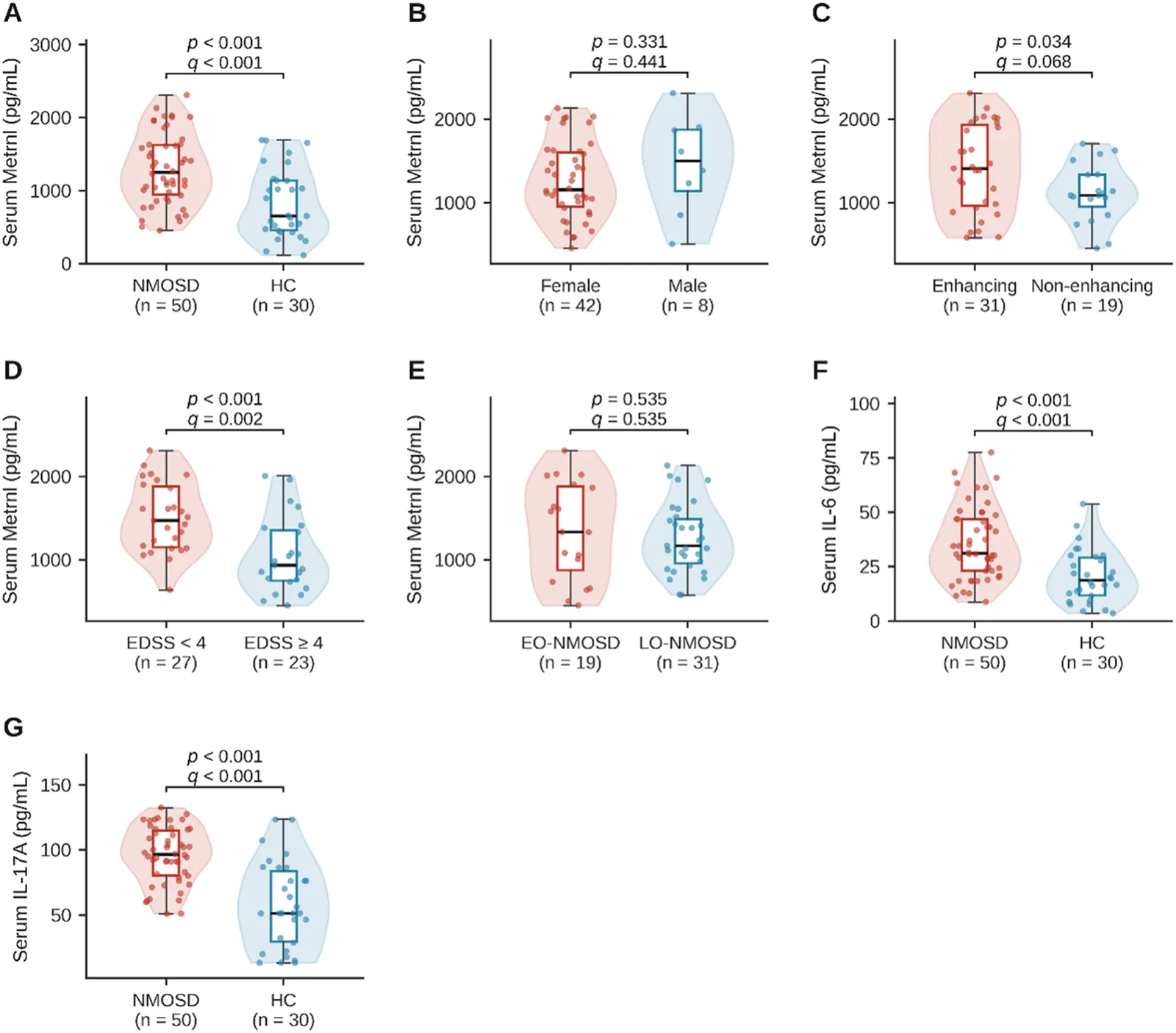
Serum levels of Metrnl and inflammatory biomarkers in patients with neuromyelitis optica spectrum disorder (NMOSD) and healthy controls (HCs). Serum levels of Metrnl **(A)**, interleukin (IL)-6 **(F)**, and IL-17A **(G)** in patients with NMOSD compared with those in HCs. **(B)** Sex-stratified serum Metrnl levels. **(C)** Serum Metrnl levels in patients with NMOSD with and without gadolinium-enhancing lesions on brain or spinal cord MRI. **(D)** Serum Metrnl levels in patients with NMOSD with mild disability (Expanded Disability Status Scale (EDSS) score < 4) versus those with moderate-to-severe disability (EDSS score ≥ 4). **(E)** Serum Metrnl levels in early-onset NMOSD (EO-NMOSD; age at disease onset < 50 years) versus late-onset NMOSD (LO-NMOSD; age at disease onset ≥ 50 years). Violin plots show the kernel density of each distribution, boxes indicate the median and interquartile range, whiskers represent 1.5 × the interquartile range; each symbol represents one participant. The *p* values are unadjusted and derived from Student’s *t*-test; *q* values are Benjamini–Hochberg false-discovery-rate adjusted *p* values within the corresponding family of tests.

Additionally, serum levels of the proinflammatory cytokines IL-6 (35.71 ± 16.61 pg/mL) and IL-17A (95.91 ± 21.65 pg/mL) were significantly greater in the acute phase in patients with NMOSD than in HCs (20.55 ± 12.19 pg/mL, *p* < 0.001; 58.34 ± 32.92 pg/mL, *p* < 0.001; both *q* < 0.001; **Figures 1F, G**). Compared with HCs, patients with NMOSD also had significantly higher platelet counts, hs-CRP levels, PLR, and SII (all *q* < 0.05; **Table 1**). The higher neutrophil count and NLR observed in the NMOSD group were nominally significant (*p* = 0.020 and *p* = 0.022, respectively) but did not remain significant after FDR correction (both *q* = 0.059). In contrast, no significant differences were observed between the two groups in WBC, monocyte, or lymphocyte counts; or in the levels of TG, TC, LDL-C, HDL-C, blood glucose, SIRI, MLR, WHR, NHR, or MHR (all *p* > 0.05; **Table 1**).

### 3.3 ROC curve analysis of serum Metrnl for NMOSD diagnosis

ROC curve analysis was employed to assess the diagnostic performance of the serum Metrnl level in distinguishing patients with NMOSD from HCs. Serum Metrnl levels exhibited statistically significant discriminative capacity, yielding an area under the ROC curve (AUC) of 0.752 (95% confidence interval [CI]: 0.642–0.842; *p* < 0.001). At the optimal cutoff value of 659.11 pg/mL, the assay achieved a sensitivity of 88.0% and a specificity of 53.3% (**Figure 2A**).

**Figure 2:**
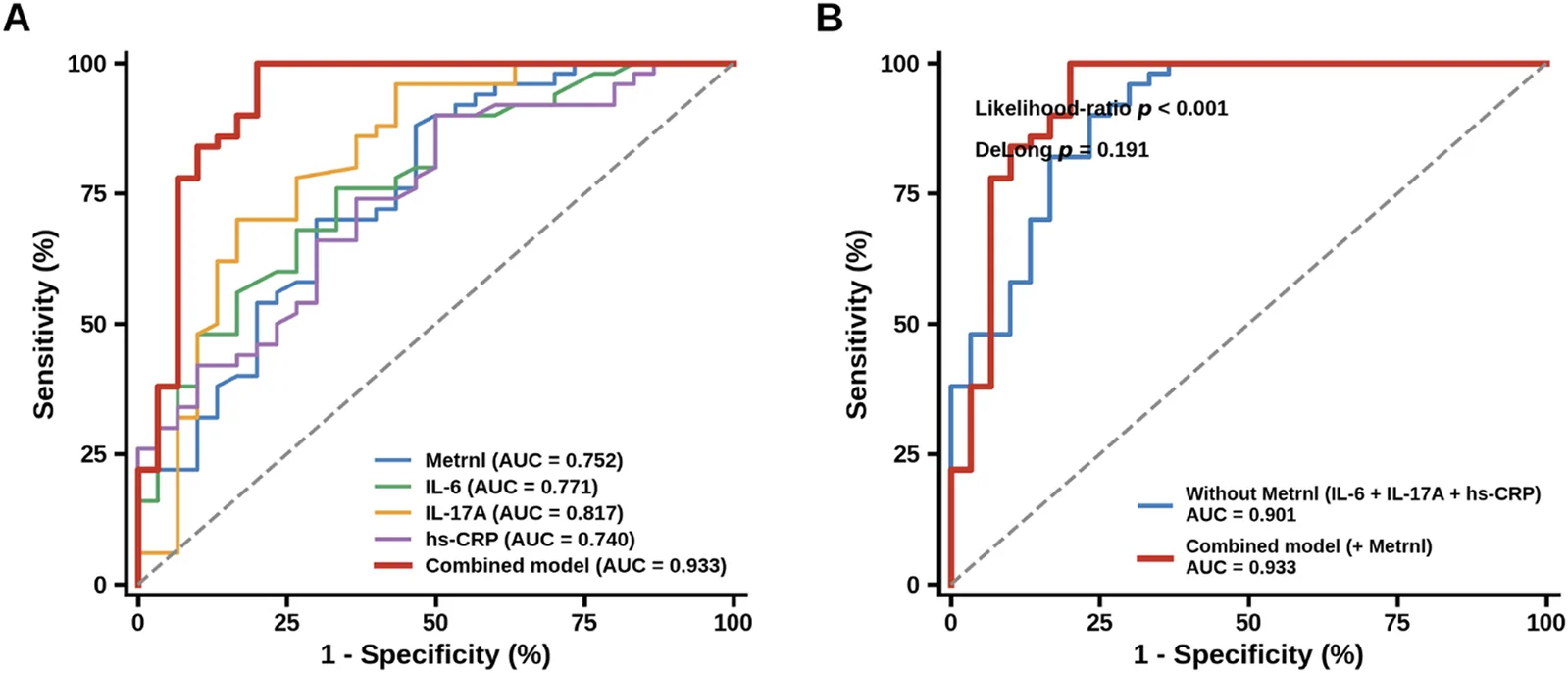
Diagnostic performance of the combined multimarker model for neuromyelitis optica spectrum disorder (NMOSD). **(A)** Receiver operating characteristic (ROC) curves for serum Metrnl, interleukin (IL)-6, IL-17A, and high-sensitivity C-reactive protein (hs-CRP) individually and for the combined logistic regression model incorporating all four biomarkers. **(B)** ROC curves of the combined model compared with those of a model containing only IL-6, IL-17A, and hs-CRP; *p* values are from the DeLong test and from the likelihood-ratio test for the addition of Metrnl. AUC, area under the curve.

### 3.4 Construction and evaluation of a combined multimarker diagnostic model

Given the limited specificity of serum Metrnl as a standalone biomarker, a multimarker diagnostic model was constructed using logistic regression. In univariable analysis, serum Metrnl, IL-6, IL-17A, hs-CRP, neutrophil count, platelet count, NLR, PLR, and SII were each significantly associated with NMOSD, whereas age was not significantly associated (**Table 2**). Because neutrophil count, NLR, PLR, and SII were strongly intercorrelated, these derived indices were not entered into the model simultaneously. Moreover, none of them retained independent significance once the cytokine panel was included; therefore, they were excluded from the final model. The final multivariable model retained serum Metrnl, IL-6, IL-17A, and hs-CRP levels, each of which remained independently associated with NMOSD (Metrnl: OR 4.45 per 1-SD increment, 95% CI 1.70–11.67, *p* = 0.002; IL-6: OR 3.57, 95% CI 1.32–9.64, *p* = 0.012; IL-17A: OR 3.99, 95% CI 1.68–9.47, *p* = 0.002; hs-CRP: OR 3.92, 95% CI 1.23–12.50, *p* = 0.021; **Table 2**).

**Table 2:** Univariable and multivariable logistic regression analyses of factors associated with NMOSD.

Variable	Crude OR (95% CI)	*p*	Adjusted OR (95% CI)	*p*	VIF
Metrnl	2.96 (1.62–5.38)	<0.001	4.45 (1.70–11.67)	0.002	1.04
IL-6	3.58 (1.77–7.24)	<0.001	3.57 (1.32–9.64)	0.012	1.15
IL-17A	4.75 (2.33–9.68)	<0.001	3.99 (1.68–9.47)	0.002	1.27
hs-CRP	3.23 (1.54–6.78)	0.002	3.92 (1.23–12.50)	0.021	1.12
NEU	2.13 (1.15–3.96)	0.016	–	–	–
PLT	2.46 (1.39–4.33)	0.002	–	–	–
NLR	2.09 (1.06–4.15)	0.034	–	–	–
PLR	3.31 (1.23–8.89)	0.018	–	–	–
SII	3.28 (1.34–8.04)	0.010	–	–	–
Age	1.00 (0.97–1.03)	0.800	–	–	–

ORs are expressed per 1-standard-deviation increment of each continuous variable. Adjusted ORs are from the multivariable model retaining Metrnl, IL-6, IL-17A, and hs-CRP. OR, odds ratio; CI, confidence interval; VIF, variance inflation factor; IL, interleukin; hs-CRP, high-sensitivity C-reactive protein; NEU, neutrophil count; PLT, platelet count; NLR, neutrophil-to-lymphocyte ratio; PLR, platelet-to-lymphocyte ratio; SII, systemic immune-inflammation index.

The predicted probability derived from this model discriminated patients with NMOSD from HCs considerably better than any single biomarker did, with AUCs of 0.933 (95% CI 0.865–1.000) versus 0.752 for Metrnl, 0.771 for IL-6, 0.817 for IL-17A and 0.740 for hs-CRP (DeLong *p* < 0.001, *p* = 0.004, *p* = 0.003 and *p* < 0.001, respectively; **Figure 2A**, **Table 3**). At the optimal cutoff (predicted probability 0.355), the combined model achieved a sensitivity of 100.0% and a specificity of 80.0%, corresponding to a Youden index of 0.800 and an overall accuracy of 92.5%; this represented a substantial increase in specificity over the 53.3% obtained with Metrnl alone. Internal validation supported the stability of the model: the bootstrap optimism-corrected AUC was 0.917, and the mean AUC across repeated fivefold cross-validation was 0.900 ± 0.012. A nomogram was constructed to facilitate the application of the model (**Figure 3A**). Calibration was adequate (Hosmer–Lemeshow *p* = 0.068; Brier score 0.080; **Figure 3B**), and decision curve analysis revealed a greater net benefit than Metrnl alone or the treat-all and treat-none strategies across threshold probabilities of approximately 0.05–0.85 (**Figure 3C**). To determine whether Metrnl contributed information beyond the conventional biomarkers, the combined model was compared with a model comprising only IL-6, IL-17A, and hs-CRP. Adding Metrnl increased the AUC from 0.901 to 0.933; however this increase did not reach statistical significance according to the DeLong test (*p* = 0.191), which is conservative for nested models at this sample size, and the addition of Metrnl significantly improved the model fit (likelihood-ratio χ² = 13.90, *p* < 0.001; AIC 56.88 versus 68.78) and significantly improved the risk reclassification (IDI 0.149, 95% CI 0.079–0.232, *p* = 0.001; continuous NRI 0.720, 95% CI 0.360–1.427, *p* = 0.001) (**Figure 2B**).

**Table 3:** Diagnostic performance of individual biomarkers and the combined model in differentiating patients with NMOSD from healthy controls.

Model	AUC (95% CI)	Cutoff	Sen	Spe	YI	Acc	*p*
Metrnl alone	0.752 (0.642–0.842)	659.11 pg/mL	88.0	53.3	0.413	75.0	Ref.
IL-6 alone	0.771 (0.666–0.876)	23.07 pg/mL	76.0	66.7	0.427	72.5	0.799
IL-17A alone	0.817 (0.712–0.923)	91.00 pg/mL	70.0	83.3	0.533	75.0	0.357
hs-CRP alone	0.740 (0.629–0.851)	1.20 mg/L	90.0	50.0	0.400	75.0	0.886
IL-6 + IL-17A + hs-CRP	0.901 (0.829–0.973)	0.512	90.0	76.7	0.667	85.0	0.021
Combined model	0.933 (0.865–1.000)	0.355	100.0	80.0	0.800	92.5	<0.001

The combined model comprises Metrnl, IL-6, IL-17A, and hs-CRP. The cutoffs for single biomarkers are given in assay units; for the two multimarker models, the cutoff is the predicted probability. *p* values are from DeLong tests versus serum Metrnl alone. For the combined model, the bootstrap optimism-corrected AUC was 0.917 (1000 resamples), and the mean AUC over 200 repetitions of fivefold cross-validation was 0.900. AUC, area under the curve; Sen, sensitivity (%); Spe, specificity (%); YI, Youden index; Acc, accuracy (%).

**Figure 3:**
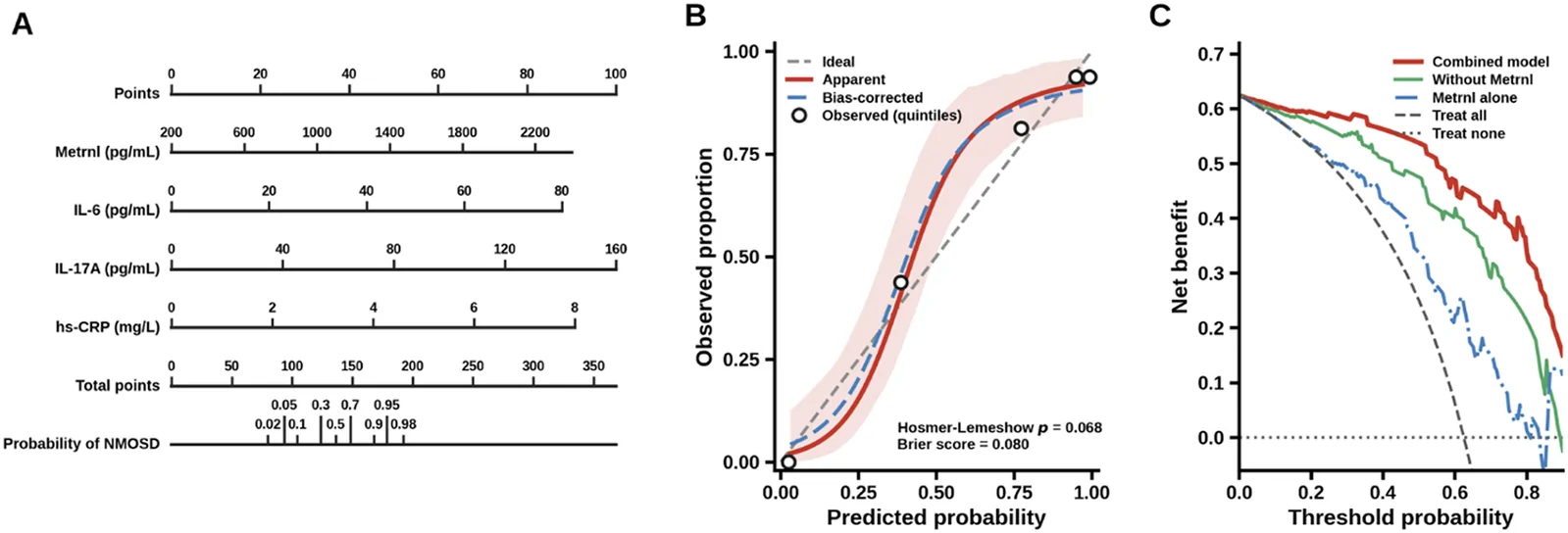
Nomogram, calibration and clinical utility of the combined multimarker model. **(A)** Nomogram derived from the multivariable logistic regression model; points are read for each biomarker, summed, and the total projected onto the probability axis. **(B)** Calibration curve showing the apparent curve, the bootstrap bias-corrected curve (1000 resamples) with its 95% confidence interval, and the observed proportions by quintile of predicted probability against the line of ideal calibration. **(C)** Decision curve analysis comparing the combined model, the model without Metrnl, Metrnl alone, and the treat-all and treat-none strategies.

### 3.5 Correlations between serum Metrnl levels and clinical or laboratory parameters in patients with NMOSD

Serum Metrnl levels in patients with NMOSD exhibited significant negative correlations with IL-6 (*r* = -0.353, *p* = 0.012, *q* = 0.034; **Figure 4A**) and IL-17A (*r* = -0.360, *p* = 0.010, *q* = 0.034; **Figure 4B**). The inverse correlation with hs-CRP (*r* = -0.297, *p* = 0.036; **Figure 4C**) did not remain significant after FDR correction (*q* = 0.084); given that the current sample (n = 50) provided 80% power only for coefficients of |*r*| ≥ 0.39, this association is reported as hypothesis-generating. Significant negative correlations were likewise observed with NLR (*r* = -0.393, *p* = 0.005, *q* = 0.023; **Figure 4D**) and SII (*r* = -0.405, *p* = 0.004, *q* = 0.023; **Figure 4E**). A robust negative correlation was also observed with EDSS score (*r* = -0.571, *p* < 0.001, *q* = 0.007; **Figure 4F**). In contrast, no significant correlation was detected between serum Metrnl levels and PLR (*r* = -0.108, *p* = 0.455). Furthermore, Metrnl levels exhibited no statistically significant correlations with any of the measured lipid parameters, including TC (*r* = 0.078, *p* = 0.589), TG (*r* = -0.188, *p* = 0.190), HDL-C (*r* = -0.163, *p* = 0.259), or LDL-C (*r* = -0.249, *p* = 0.082), nor with fasting blood glucose levels (*r* = -0.018, *p* = 0.902). Similarly, no significant correlations were identified with clinical disease characteristics, including the number of relapses (*r* = 0.018, *p* = 0.899), disease duration (*r* = 0.093, *p* = 0.521) or annualized relapse rate (*r* = -0.099, *p* = 0.494).

**Figure 4:**
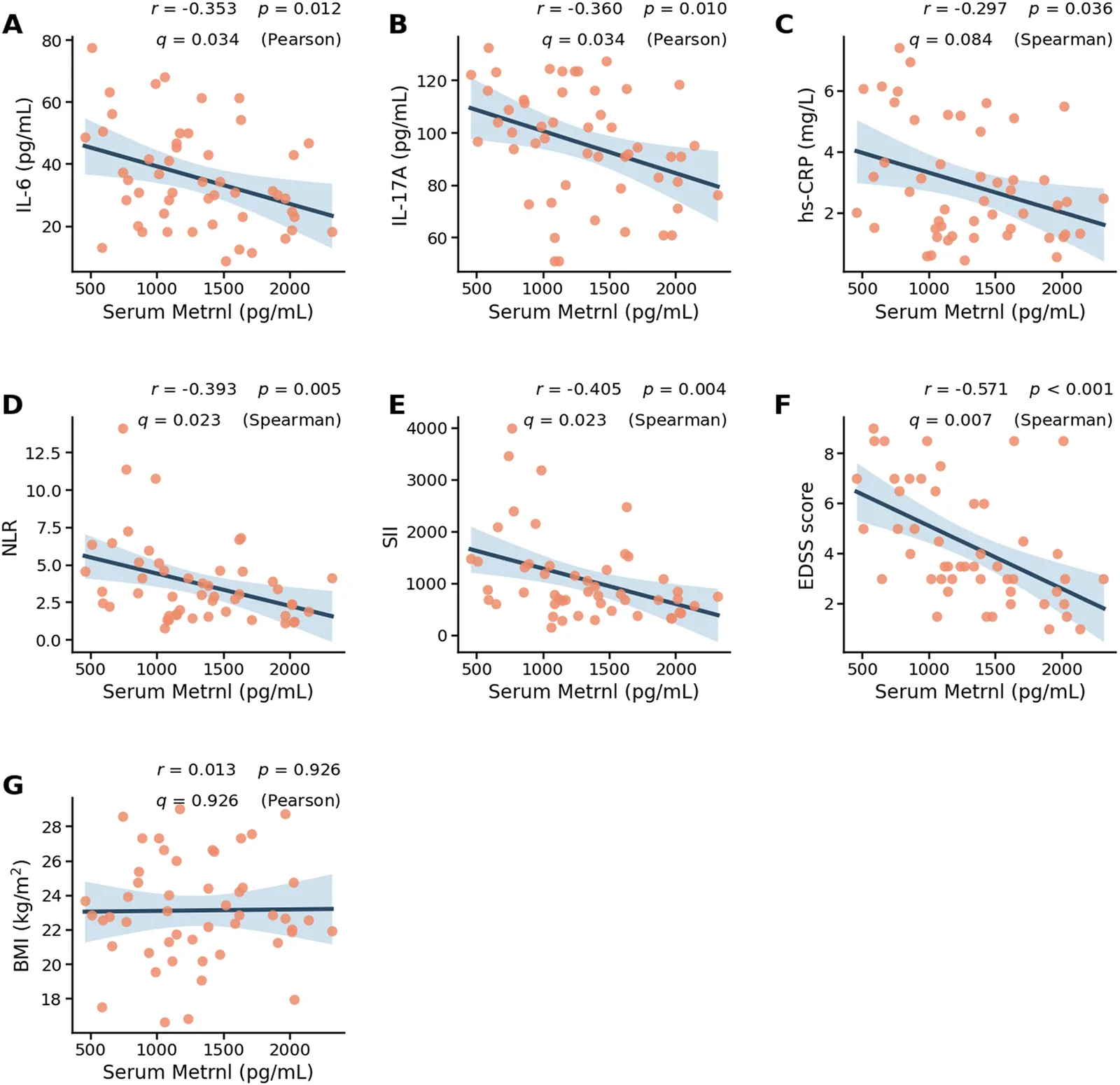
Correlations between serum Metrnl levels and clinical or laboratory parameters in patients with neuromyelitis optica spectrum disorder (NMOSD). Scatter plots depicting the associations of serum Metrnl levels with **(A)** interleukin (IL)-6, **(B)** IL-17A, **(C)** high-sensitivity C-reactive protein (hs-CRP), **(D)** neutrophil-to-lymphocyte ratio (NLR), **(E)** systemic immune-inflammation index (SII), **(F)** Expanded Disability Status Scale (EDSS) score, and **(G)** body mass index (BMI). Solid lines represent the ordinary least-squares fit, and shaded areas represent the corresponding 95% confidence band; each symbol represents one patient. The correlation coefficients are Pearson’s *r* for normally distributed variables and Spearman’s rank correlation coefficient otherwise, as indicated in each panel. *q* values are Benjamini–Hochberg false-discovery-rate-adjusted *p* values across the 15 correlations tested; only the association with hs-CRP **(C)** did not remain significant after adjustment.

Serum Metrnl levels were not correlated with BMI (Pearson *r* = 0.013, *p* = 0.926, *q* = 0.926; **Figure 4G**). To determine whether BMI confounded the association between serum Metrnl and disability, a multivariable linear regression model was fitted with serum Metrnl as the dependent variable and EDSS score, BMI, age, and sex as independent variables. The EDSS score remained independently associated with serum Metrnl (*β* = −109.24 pg/mL per EDSS point, 95% CI −164.58 to −53.90, *p* < 0.001), whereas BMI (*β* = 2.03, 95% CI −40.05 to 44.10, *p* = 0.923), age (*β* = −1.20, 95% CI −9.65 to 7.25, *p* = 0.776) and sex (*β* = −45.53 for male versus female, 95% CI −389.37 to 298.31, *p* = 0.791) were not (R^2^ = 0.276). Consistent with this, the partial correlation between serum Metrnl and EDSS score after adjustment for BMI was −0.523 (*p* < 0.001), essentially identical to the unadjusted Pearson coefficient of −0.523, and −0.510 (*p* < 0.001) after further adjustment for age and sex.

### 3.6 Longitudinal changes in serum levels of Metrnl and other inflammatory biomarkers following immunotherapy

To investigate the dynamic modulation of serum Metrnl levels during NMOSD disease activity, paired serum samples were collected from 30 patients with NMOSD during the acute phase (both before and after high-dose methylprednisolone therapy). Neurological disability significantly improved after treatment, as reflected by a substantial reduction in EDSS score relative to baseline (3.33 ± 2.05 vs. 4.27 ± 2.62 at pretreatment; *p* < 0.001, *q* = 0.002; **Table 4**). Serum Metrnl levels significantly decreased following treatment (999.82 ± 313.78 pg/mL vs. 1390.48 ± 501.87 pg/mL at pretreatment; *p* < 0.001, *q* < 0.001, *d*_z_ = 1.45) (**Figure 5A**). Serum IL-6 (31.26 ± 13.16 vs. 34.89 ± 13.98 pg/mL) and IL-17A (93.54 ± 19.18 vs. 97.31 ± 20.31 pg/mL) also declined significantly after treatment (both *p* < 0.001 and *q* < 0.001; **Figures 5B, C**). The relative magnitude of the treatment-associated change differed markedly between biomarkers: the median decrease from baseline was 25.5% for Metrnl, compared with 10.2% for IL-6, 2.9% for IL-17A and less than 4% for NLR, PLR, and SII (**Figure 5D**). In contrast, no significant differences were observed in the NLR (*p* = 0.343), PLR (*p* = 0.236), or SII (*p* = 0.186) following treatment (**Figures 5E–G**; **Table 4**). Importantly, the within-patient changes in serum Metrnl levels did not correlate with the concurrent changes in IL-6 (*r* = -0.088, *p* = 0.644), IL-17A (*r* = -0.009, *p* = 0.964), NLR (*r* = 0.143, *p* = 0.452), PLR (*r* = 0.077, *p* = 0.685) or SII (*r* = 0.063, *p* = 0.741) (**Figure 5H**). With 30 paired observations, coefficients of |*r*| ≥ 0.49 would have been detectable with 80% power; thus, only strong dynamic coupling between Metrnl and these biomarkers could be excluded. All longitudinal comparisons are summarized in **Table 4**.

**Table 4:** Longitudinal changes in disability and circulating inflammatory biomarkers before and after high-dose methylprednisolone pulse therapy in 30 patients with NMOSD.

Variable	Before treatment	After treatment	Median change (%)	*p*	*q*
Serum Metrnl (pg/mL)	1390.48 ± 501.87	999.82 ± 313.78	−25.5	<0.001	<0.001
EDSS score	4.27 ± 2.62	3.33 ± 2.05	–	<0.001	0.002
Serum IL-6 (pg/mL)	34.89 ± 13.98	31.26 ± 13.16	−10.2	<0.001	<0.001
Serum IL-17A (pg/mL)	97.31 ± 20.31	93.54 ± 19.18	−2.9	<0.001	<0.001
NLR, median (IQR)	3.14 (1.67–4.59)	3.18 (1.74–4.60)	−0.9	0.343	0.393
PLR, median (IQR)	163.76 (119.39–206.15)	159.46 (116.30–200.15)	−2.2	0.236	0.316
SII, median (IQR)	805.43 (434.64–1275.02)	804.17 (497.14–1267.73)	−3.3	0.186	0.316

Values are the mean ± standard deviation for normally distributed variables and the median (interquartile range) for nonnormally distributed variables, as indicated in the first column. The median change (%) is the median within-patient percentage change from baseline; it is not reported for the EDSS because that scale is ordinal (mean absolute reduction 0.94 points). *p* values are from paired *t*-tests or Wilcoxon signed-rank tests, as appropriate; *q* values are Benjamini–Hochberg false-discovery-rate-adjusted *p* values across the seven paired comparisons of this family. Changes in serum Metrnl levels were not correlated with concurrent changes in IL-6 levels, IL-17A levels, the NLR, the PLR, or the SII (Spearman |*r*| ≤ 0.14, all *p* > 0.4). EDSS, Expanded Disability Status Scale; IL, interleukin; NLR, neutrophil-to-lymphocyte ratio; PLR, platelet-to-lymphocyte ratio; SII, systemic immune-inflammation index.

**Figure 5:**
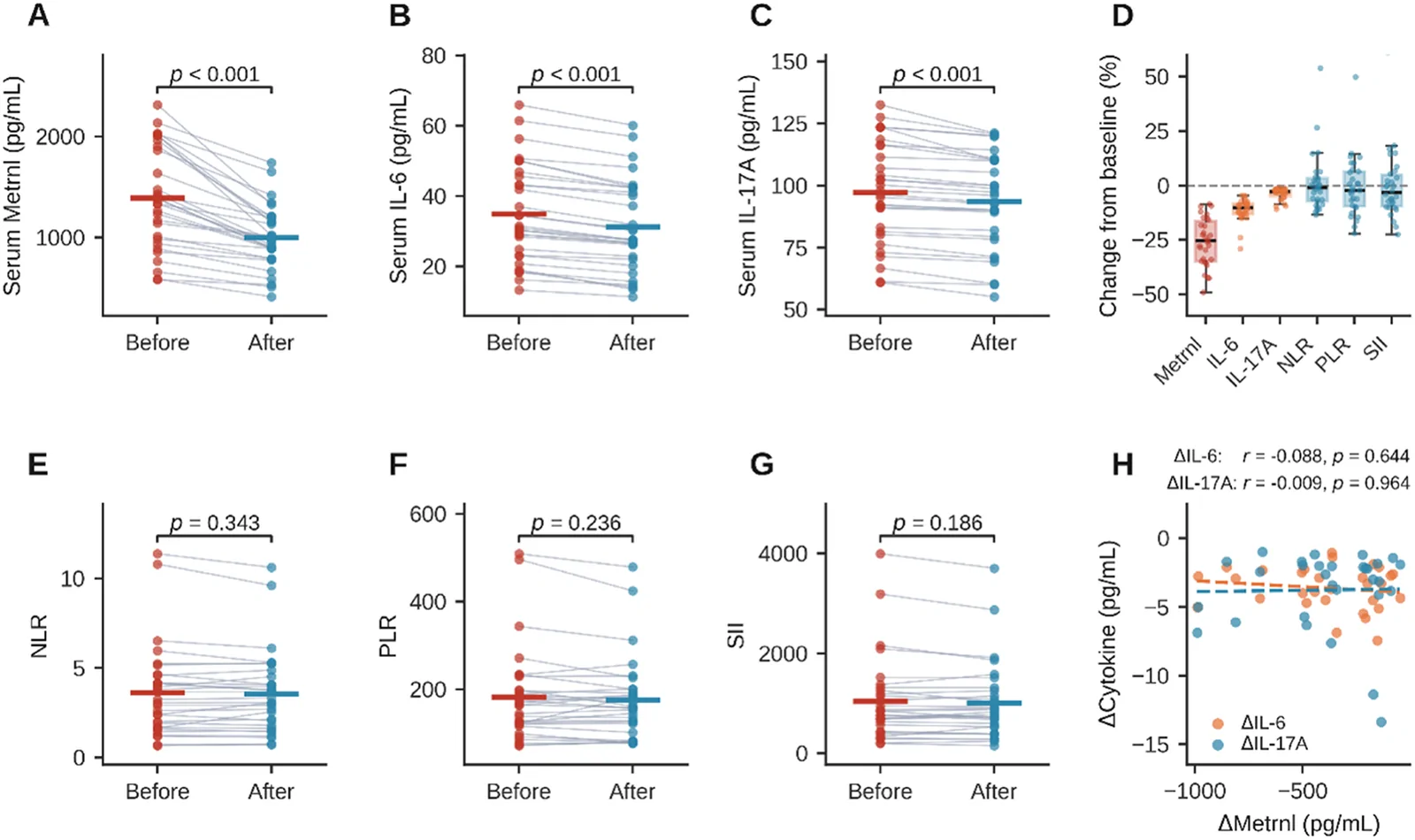
Longitudinal changes in serum levels of Metrnl and other inflammatory biomarkers following immunotherapy in patients with neuromyelitis optica spectrum disorder (NMOSD). Paired serum levels of **(A)** Metrnl, **(B)** interleukin (IL)-6, and **(C)** IL-17A, and paired values of **(E)** neutrophil-to-lymphocyte ratio (NLR), **(F)** platelet-to-lymphocyte ratio (PLR), and **(G)** systemic immune-inflammation index (SII) in 30 patients with NMOSD before and after high-dose methylprednisolone pulse therapy; thin gray lines connect the two measurements from the same patient and horizontal bars indicate group means. **(D)** Percentage change from baseline for each biomarker; the dashed line indicates no change. **(H)** Relationships between the within-patient changes in serum Metrnl levels and the concurrent changes in IL-6 and IL-17A levels, with the corresponding Spearman rank correlation coefficients; the dashed lines show the least-squares fits. The *p* values in **(A–C, E–G)** are from paired *t*-tests or Wilcoxon signed-rank tests, as appropriate.

## 4 Discussion

In this study, serum Metrnl levels were significantly higher in patients with NMOSD than in HCs. Stratified analysis suggested that patients with MRI-detectable gadolinium-enhancing lesions had higher serum Metrnl levels than did those without such lesions, although this difference was nominal and did not survive correction for multiple comparisons. Moreover, serum Metrnl levels were significantly and negatively correlated with IL-6 levels, IL-17A levels, NLR, SII, and EDSS score, indicating significant associations with both systemic inflammatory activity and neurological disability severity. Longitudinal assessment further revealed that serum Metrnl levels significantly decreased following high-dose intravenous methylprednisolone therapy, in parallel with reductions in IL-6 and IL-17A levels, thereby supporting the dynamic responsiveness of this parameter to acute immunomodulatory intervention. ROC curve analysis demonstrated that the serum Metrnl had significant diagnostic utility for distinguishing patients with NMOSD from HCs. Collectively, these findings indicate that Metrnl represents a promising candidate biomarker for elucidating NMOSD pathophysiology, monitoring disease activity, and evaluating therapeutic response.

NMOSD is a prototypical autoantibody-mediated autoimmune disease of the CNS, whose pathogenesis is primarily attributable to systemic immune dysregulation (25). Accumulating evidence indicates that aberrant differentiation and functional imbalance among CD4^+^ T-cell subsets—particularly the expansion and hyperactivation of Th17 and follicular helper T cells, in conjunction with diminished suppressive function or a reduced frequency of regulatory T cells— represent central immunopathogenic mechanisms in NMOSD (26, 27). Furthermore, proinflammatory cytokines and downstream inflammatory mediators critically contribute to neuroinflammation, BBB disruption, and astrocyte injury in NMOSD (28, 29).

Metrnl, a recently characterized cytokine with potent immunomodulatory properties, has emerged as a key regulator of both innate and adaptive immune responses (15). It is predominantly produced by functionally distinct macrophage subsets and is robustly expressed in barrier tissues (e.g., skin and mucosal epithelia), as well as in activated monocytes (15). Notably, dysregulated Metrnl expression has been consistently observed across diverse autoimmune disorders, including psoriasis, inflammatory bowel disease (IBD), RA, and Graves’ disease (GD) (13). In patients with RA, elevated Metrnl levels have been reported in both serum and synovial tissue, indicating concurrent systemic and local inflammatory involvement, whereas in patients with GD, circulating Metrnl levels are significantly suppressed (17, 30, 31). Similarly, reduced peripheral Metrnl levels have been reported in Crohn’s disease (CD) and ulcerative colitis (UC) cohorts compared with those in HCs; however, Metrnl expression has been paradoxically upregulated in the mesenteric adipose tissue of patients with CD (32, 33). Collectively, these findings suggest that Metrnl may participate in the pathological processes of different autoimmune diseases through multiple mechanisms. Nevertheless, its role in NMOSD remains uncharacterized. In the present study, serum Metrnl levels were significantly elevated in patients with NMOSD compared with HCs. Stratified analysis further indicated that patients with MRI-detectable gadolinium-enhancing lesions had higher serum Metrnl levels than those without such lesions; because this comparison did not survive FDR correction and was underpowered, it is best regarded as a preliminary signal that Metrnl might track radiological disease activity, and requires confirmation in a larger, prospectively defined cohort. ROC curve analysis demonstrated that serum Metrnl had significant diagnostic utility for distinguishing patients with NMOSD from HCs, thereby supporting its potential as a clinically translatable auxiliary diagnostic biomarker.

Although the diagnostic performance of serum Metrnl as an independent biomarker remains moderate—with an area under the curve (AUC) of 0.752 and a specificity of only 53.3% at the optimal cutoff value—these findings are insufficient to support its use as an independent diagnostic test. Therefore, we employed a logistic regression approach to construct a combined model. When Metrnl was analyzed in conjunction with IL-6, IL-17A, and hs-CRP, the AUC increased to 0.933; this combined model increased the specificity from 53.3% to 80.0% while maintaining a sensitivity of 100%, thereby significantly reducing the misclassification rate in healthy individuals. These results indicated that Metrnl should not be regarded as an independent diagnostic test but rather as a supplementary component within a multibiomarker panel. These findings should also be interpreted within the broader biomarker-discovery landscape. Comprehensive serum metabolic fingerprinting by nanoparticle-enhanced laser desorption/ionization mass spectrometry has been reported to distinguish patients with NMOSD from controls with AUC values exceeding 0.90 and to identify bile acid-related discriminants (34); comparable progress has been achieved with ultrasensitive blood-based phosphorylated tau assays in Alzheimer’s disease (35) and with single-cell and spatially resolved profiling of organ-specific fibro-inflammatory disease (36). Relative to these platforms, an enzyme-linked immunosorbent assay panel of four circulating proteins is inexpensive, technically undemanding, and readily transferable to routine clinical laboratories, but it cannot match the breadth of information provided by untargeted omics approaches. We therefore position Metrnl not as a competitor to such technologies but as an accessible and mechanistically informative component of a compact multimarker panel, which will itself require head-to-head comparison with omics-based signatures in independent cohorts.

This study further investigated the associations between serum Metrnl levels and systemic inflammatory parameters. The results revealed statistically significant negative correlations between Metrnl levels and NLR and SII values, as well as the levels of IL-6, IL-17A, and hs-CRP. Systemic inflammatory indices (including NLR, PLR, and SII) have been established as robust prognostic indicators across multiple autoimmune disorders (37–39). Notably, elevated NLR and SII values have been consistently reported in patients with NMOSD and are strongly associated with disease activity and poor clinical outcomes (40, 41). Thus, the inverse relationship between serum Metrnl levels and these validated inflammatory metrics supports the potential role of Metrnl as a novel modulator within the inflammatory regulatory network underlying NMOSD pathogenesis and progression.

IL-6 and IL-17A are pivotal cytokines involved in the immunopathogenesis of NMOSD. IL-6 promotes the differentiation of autoreactive B cells into antibody-secreting plasmablasts and enhances pathogenic anti-AQP4 antibody production, thereby establishing itself as a central driver of NMOSD pathophysiology (28). Clinically, blockade of the IL-6 signaling pathway with monoclonal antibodies targeting the IL-6 receptor has demonstrated robust efficacy in reducing annualized relapse rates and stabilizing neurological disability in patients with NMOSD, thus underscoring the therapeutic relevance of this axis (42, 43). IL-17A, which is predominantly secreted by Th17 cells, contributes to BBB disruption, neutrophil infiltration, and astrocyte injury within the CNS (11, 44). Elevated IL-17A levels have been consistently documented in both the serum and cerebrospinal fluid of patients with NMOSD and are significantly correlated with clinical disease activity (20). The statistically significant inverse correlations observed between serum Metrnl levels and both IL-6 and IL-17A levels in this study are consistent with the hypothesis that Metrnl counterbalances the proinflammatory milieu characteristic of NMOSD. This hypothesis is supported by experimental evidence indicating that in cell-based and animal models, Metrnl reduces IL-6 and IL-17A expression through the STAT5/PPAR-γ and AMPK–PAK2 signaling pathways (33, 45, 46); such mechanistic inferences derive from interventional systems and cannot be transferred directly to the cross-sectional associations reported here. Furthermore, Metrnl has been observed to inhibit T-cell proliferation, and its expression is induced by the Th2-type cytokines IL-4 and IL-13 (47), which may indirectly attenuate Th17 differentiation and, consequently, IL-17A production. In Metrnl-deficient mice, pronounced inflammatory phenotypes with elevated tumor necrosis factor-α levels further corroborate the immunosuppressive function of this cytokine (16). Moreover, the inverse association between Metrnl and hs-CRP aligns with findings reported in individuals with GD (31), thus reinforcing the hypothesis that Metrnl exerts systemic anti-inflammatory effects, although in the present cohort this particular association did not survive correction for multiple comparisons. In MG, Metrnl levels are positively correlated with CD19^+^ B-cell counts but negatively correlated with plasma cell counts, suggesting that Metrnl has a regulatory effect on B-cell subset homeostasis (18). Given that dysregulated T-cell differentiation constitutes a critical mechanism driving autoantibody production (48) and considering the ability of Metrnl to modulate key inflammatory cytokines and immune cell dynamics (33, 45, 46), we propose that Metrnl may participate in the development and progression of NMOSD by modulating cytokine networks to influence T-cell proliferation, Th17 differentiation, and B-cell maturation. Although the specific functions of Metrnl remain to be fully elucidated, the available evidence is compatible with a predominantly counter-regulatory role in this setting, while the human data remain associative.

Two aspects of this interpretation warrant explicit caution. First, the literature portrays Metrnl inconsistently: circulating and synovial levels are elevated in RA and correlate positively with disease activity (17, 30), whereas they are reduced in GD (31) and in IBD (32, 33); experimental work has demonstrated that both proinflammatory (19) and anti-inflammatory (16, 45–47) actions depend on the cell type, tissue compartment, species, and stimulus examined. Rather than reflecting a fixed proinflammatory or anti-inflammatory identity, these observations are most consistent with context-dependent functionality, in which the net effect of Metrnl varies with the local cytokine milieu, the compartment sampled, and the stage of the inflammatory response; peripheral blood levels may therefore diverge from tissue-level activity, as is directly demonstrated by the discordance between circulating and mesenteric adipose Metrnl in patients with CD (33). Second, the associations reported here are cross-sectional and correlational, and the direction of the relationships between Metrnl and IL-6, IL-17A, hs-CRP, NLR, or SII cannot be established. Reverse causality is equally plausible: sustained proinflammatory signaling may deplete or exhaust Metrnl-producing myeloid populations rather than Metrnl suppressing cytokine production. Confounding by disease duration, cumulative relapse burden, and prior immunotherapy cannot be excluded, and no multivariable adjustment was applied to the correlation analyses; the absence of a dynamic correlation between changes in Metrnl and changes in IL-6 or IL-17A after treatment further cautions against inferring a direct regulatory link from these data.

Moreover, serum Metrnl levels significantly decreased following treatment in 30 patients with NMOSD, and this decline temporally coincided with marked clinical improvement. Both direct and indirect mechanisms could account for this observation. Glucocorticoids act genomically through the glucocorticoid receptor, which can transactivate genes bearing glucocorticoid-response elements and, more commonly for inflammatory genes, transrepress NF-κB- and AP-1-driven transcription. Because Metrnl expression in bone marrow-derived and barrier-tissue cells is induced by proinflammatory stimuli that act largely through NF-κB (16, 19), methylprednisolone could lower circulating Metrnl either by direct transcriptional repression of the Metrnl gene or, indirectly, by extinguishing the inflammatory signals that drive its compensatory upregulation. Several observations favor the indirect route: serum IL-6 and IL-17A levels decreased in parallel with those of Metrnl, the decrease in Metrnl accompanied clinical improvement rather than deterioration, and the proportional decrease in Metrnl (median 25.5%) was considerably greater than that of IL-6 (10.2%) or IL-17A (2.9%), which is difficult to reconcile with simple co-suppression of a shared transcriptional program. Conversely, the complete absence of any within-patient correlation between the change in Metrnl and the concurrent changes in IL-6, IL-17A, or the cell-count-derived indices argues against a tightly coupled, stoichiometric relationship between Metrnl and these mediators, and the two mechanisms cannot be distinguished from clinical data alone. Because no untreated NMOSD comparator group was sampled over the same interval, spontaneous resolution of the acute attack cannot be separated from a pharmacological effect. Promoter- and chromatin-level studies of Metrnl regulation under glucocorticoid exposure, together with dose- and time-resolved sampling, will be needed to answer this question. Although preliminary in nature, these findings provide novel mechanistic insights into the potential role of Metrnl in the immunopathogenesis of NMOSD.

Furthermore, although serum Metrnl levels were significantly higher in patients with NMOSD than in HCs, they were inversely correlated with EDSS scores. These seemingly paradoxical findings reflect the dynamic, biphasic trajectory of Metrnl throughout the disease course. We propose that the low level of circulating Metrnl in healthy individuals represents a physiologic homeostatic baseline, whereas the relatively low level of Metrnl observed in severely disabled patients reflects the pathologic depletion of secretory reservoirs following repeated relapses and sustained inflammation. This observation leads us to propose a “compensatory exhaustion hypothesis”: during acute inflammation, innate immune cells upregulate Metrnl as a negative feedback mechanism to counteract IL-6/IL-17A, resulting in compensatory elevation of serum Metrnl; however, as the disease progresses chronically, exhaustion of Metrnl-producing cells leads to declining levels, loss of anti-inflammatory buffering, unopposed pathogenic cytokine activity, and accelerated neural injury and disability accumulation. This framework is supported by our findings, including the inverse correlation between Metrnl and EDSS score, the negative correlations with IL-6 and IL-17A, and the observation that post-treatment Metrnl decline paralleled clinical improvement—suggesting that the reduction reflects diminished inflammatory demand rather than therapeutic suppression of synthesis. Similar tissue-specific biphasic patterns have been reported in IBD and RA (17, 30, 32, 33), highlighting the importance of interpreting biomarker levels within the clinical context of disease chronicity. Nevertheless, this hypothesis requires validation through prospective longitudinal studies with serial sampling across acute attacks, remission, and chronic progression phases. As set out below, this framework also carries a more fundamental limitation: it accommodates both a decrease and an increase in serum Metrnl, and therefore cannot explain the discordance between our cross-sectional and longitudinal findings.

The divergent behavior of serum Metrnl with respect to the two indices of severity used here follows from this framework. Gadolinium enhancement is a state marker: it reflects focal BBB breakdown and inflammatory activity at the moment of imaging and resolves within weeks of an attack. In contrast, the EDSS score is a cumulative measure that aggregates the residual deficits left by every previous attack; because disability in AQP4-IgG-positive NMOSD accrues almost entirely as relapse-associated worsening with incomplete recovery rather than through insidious progression (23), a high EDSS score identifies patients with a history of severe or poorly recovering attacks rather than those with the most intense inflammation at the time of sampling. The two measures therefore capture the acute and the chronic ends of the trajectory outlined above, and Metrnl would be expected to behave differently with respect to each: higher in the presence of active enhancing lesions, and lower once repeated attacks have both accumulated disability and depleted the compensatory reservoir. Consistent with these findings, serum Metrnl levels were unrelated to disease duration, the total number of attacks, and the annualized relapse rate, all of which index cumulative disease burden rather than the current inflammatory state or the severity of residual injury. This interpretation nevertheless remains provisional: the enhancing-lesion comparison did not survive correction for multiple comparisons, and only a design in which imaging, disability, and serum Metrnl are captured serially across attack and remission can establish whether the two markers genuinely dissociate in the manner proposed. One alternative explanation of the Metrnl–EDSS association requires separate consideration. Metrnl is also an adipokine, and its circulating concentration has been related to adiposity, skeletal muscle mass, and physical activity (13, 49, 50); because patients with an EDSS score of 4.0 or above have restricted ambulation by definition, the association could in principle reflect body composition rather than immune activity. The data do not support that interpretation. BMI was comparable between patients and controls, was not correlated with serum Metrnl, and altered neither the regression coefficient nor the partial correlation of the Metrnl–EDSS relationship when entered as a covariate alongside age and sex. BMI is nonetheless a crude proxy that does not separate fat from lean mass, so this explanation is weakened rather than excluded, and direct measurement of body composition and of habitual physical activity would be needed to exclude it.

That discordance deserves to be stated plainly rather than resolved by hypothesis alone. Across patients, serum Metrnl decreased as EDSS score increased, with a large difference between the disability strata (Cohen’s d = 1.05); within patients, methylprednisolone produced the largest proportional decrease of any marker measured (median −25.5%) at the same time as disability improved. If the cross-sectional gradient described a relationship that also held within individuals, clinical improvement should have been accompanied by a rise in serum Metrnl. The compensatory exhaustion framework does not remove this incompatibility, because it attributes the low levels of severely disabled patients to exhausted production and the post-treatment decrease to diminished inflammatory demand, two distinct mechanisms invoked for two opposite directions of change. If applied consistently, this reasoning would also predict lower serum Metrnl in the mild-disability group, in whom inflammatory activity was lowest, yet that group had the highest levels. We therefore retain that framework as a conjecture about mechanism and withdraw it as an account of the discordance itself.

The discordance nevertheless has a straightforward formal basis. An association estimated between patients at a single time point and an association estimated within patients across time are distinct parameters that carry no requirement to share a sign, and the two are routinely separated in longitudinal analysis precisely because they can diverge (51, 52). The cross-sectional gradient compares individuals who differ in attack history and in accumulated injury as well as in current inflammatory activity, and EDSS indexes cumulative residual deficit rather than inflammatory intensity at the moment of sampling, whereas the post-treatment change holds the individual constant. Read in this way, serum Metrnl tracks acute inflammatory activity within a patient, whereas the between-patient gradient reflects something that these data cannot identify. One observation argues against the simplest demand-driven reading: the within-patient decrease in Metrnl was uncorrelated with the concurrent decreases in IL-6 and IL-17A (all |r| ≤ 0.14). Serial sampling across attack and remission, and repeated measurement across successive attacks, would distinguish these possibilities.

This study has several limitations. First, the single-center design, relatively small sample size, homogeneous ethnic background, and limited follow-up duration may limit the generalizability and external validity of the results. Further multicenter, prospective, large-cohort studies with prolonged follow-up and diverse populations are required to validate these findings. Second, the cross-sectional design precluded analysis of dynamic Metrnl expression across different disease activity states, and thus the temporal relationship between Metrnl levels and disease activity could not be determined. In addition, the exact molecular mechanisms by which Metrnl regulates NMOSD pathogenesis, including its immunomodulatory roles and downstream signaling pathways, remain to be clarified through further *in vitro* and *in vivo* mechanistic studies. Third, the diagnostic model was developed and internally validated using a moderate-sized single-center cohort with a limited observation-to-variable ratio. Although bootstrap resampling and cross-validation were applied, potential overfitting and overestimation of diagnostic performance cannot be excluded. External validation in independent prospective cohorts and disease control groups is necessary before clinical application. Fourth, several significant associations identified in the primary analysis did not persist after rigorous multiple-testing correction and should be considered exploratory. No formal *a priori* sample size calculation was performed, and *post-hoc* power analysis revealed insufficient statistical power for subgroup comparisons and weak correlation analyses. Accordingly, negative subgroup findings should be interpreted as inconclusive rather than definitive. Fifth, only HCs were enrolled, whereas patients with clinically similar inflammatory demyelinating diseases, such as multiple sclerosis and myelin oligodendrocyte glycoprotein antibody-associated disease (53), were not included. Therefore, this study supports only the ability of Metrnl to distinguish NMOSD from healthy status but cannot verify its value for differential diagnosis among demyelinating disorders. Sixth, relapse assessments were retrospective and indirect. Given the lack of systematic recording of relapse timelines and prospective follow-up data, the predictive value of serum Metrnl for future disease relapses remains undetermined. Longitudinal studies with standardized relapse monitoring are warranted. Seventh, detailed information regarding long-term maintenance immunotherapy was not collected, restricting the analysis of treatment effects on circulating Metrnl levels. Furthermore, all patients received identical acute methylprednisolone pulse therapy, precluding cross-regimen comparison. Only short-term post-treatment changes were analyzed, and the association between Metrnl and long-term therapeutic responses could not be evaluated. Finally, body composition and habitual physical activity were not measured. Although BMI did not confound the association between serum Metrnl and disability, it does not separate fat from lean mass, and a contribution of reduced muscle mass in patients with restricted ambulation cannot be formally excluded.

## 5 Conclusion

In summary, serum Metrnl levels were significantly higher in patients with NMOSD than in HCs, with a further exploratory increase in individuals with gadolinium-enhancing lesions that did not survive correction for multiple comparisons. Moreover, serum Metrnl levels exhibited significant negative correlations with clinical disease severity and multiple systemic inflammatory biomarkers, including NLR, SII, IL-17A, and IL-6. Notably, immunotherapy induced a substantial reduction in serum Metrnl levels, in parallel with significant decreases in IL-6 and IL-17A levels, supporting the potential utility of Metrnl as a dynamic biomarker of NMOSD disease activity. Future mechanistic studies are warranted to elucidate the precise role of Metrnl in NMOSD immunopathogenesis, thereby advancing our understanding of its pathophysiology and informing the rational design of targeted immunomodulatory interventions. Although Metrnl alone provided only moderate diagnostic discrimination, a logistic regression model combining Metrnl with IL-6, IL-17A, and hs-CRP performed substantially better (AUC 0.933; specificity 80.0% at 100% sensitivity), indicating that Metrnl is best used as a component of a multimarker diagnostic panel that now requires prospective external validation.

## Data Availability

The original contributions presented in the study are included in the article/Supplementary Material. Further inquiries can be directed to the corresponding author.
